# Corrigendum to “miR-331-3p Inhibits Proliferation and Promotes Apoptosis of Nasopharyngeal Carcinoma Cells by Targeting elf4B-PI3K-AKT Pathway”

**DOI:** 10.1177/1533033820963463

**Published:** 2020-10-01

Xuefang Z, Ruinian Z, Liji J, et al. miR-331-3p Inhibits Proliferation and Promotes Apoptosis of Nasopharyngeal Carcinoma Cells by Targeting elf4B-PI3K-AKT Pathway. *Technology in Cancer Research & Treatment*. 2020;19: 1-8. doi: 10.1177/1533033819892251

The published version of Figure 2 is incorrect. The correct version of the figure is as given below:

**Figure 2.**
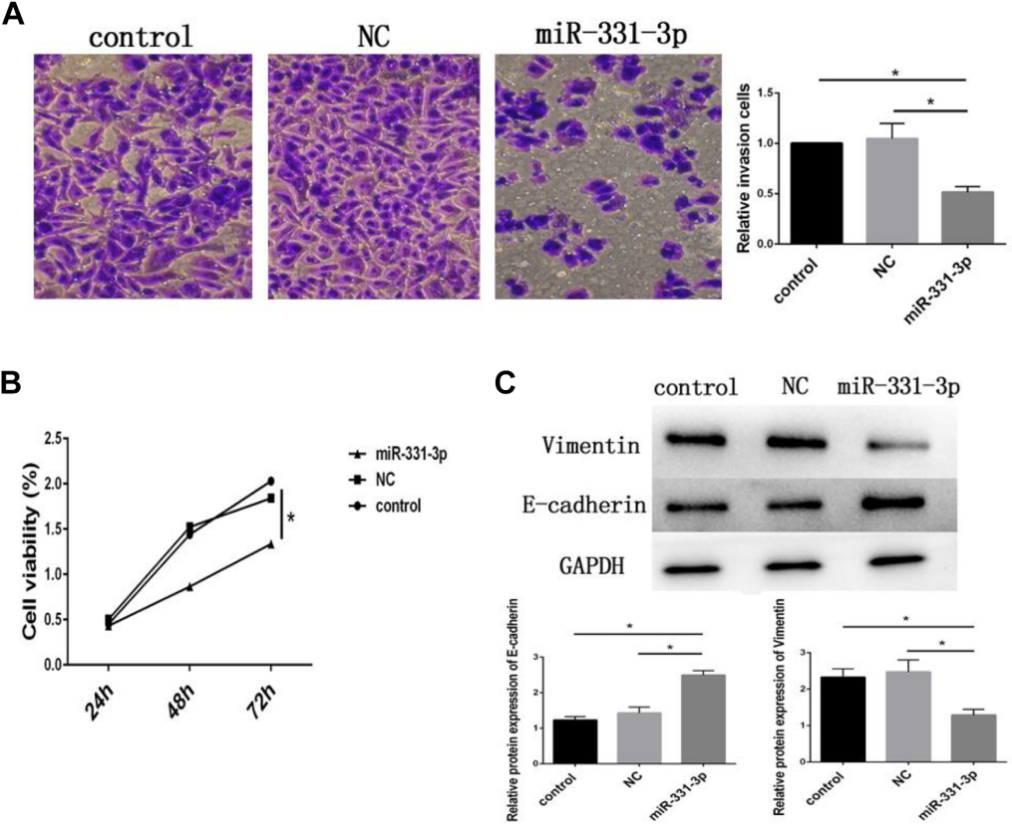
miRNA-331-3p inhibits the proliferation and invasion of CNE-1 cells. CNE-1 cells were transfected with microRNA-331-3p mimic, and those transfected with small interfering RNA duplexes with nonspecific sequences were used as the negative control (NC). A, Cell invasion was detected using Transwell invasion assay. B, Cell viability was measured using Cell Counting Kit-8 (CCK-8) assay at 0, 24, 48, and 72 hours of culture. C, The protein expression of vimentin and E-cadherin was detected by Western blot. Data are shown as the means ± standard deviations. *P < .05, compared to control or NC.

